# Review of the South East Essex Functional Intensive Response Team (First) as an Admission Avoidance Service for Older Adults

**DOI:** 10.1192/bjo.2026.11527

**Published:** 2026-06-30

**Authors:** Aderopo Adelola, Christopher St Hill, Evans Salawe, Charlotte Castledine, Feena Sebastian

**Affiliations:** Essex Partnership University NHS Foundation Trust, Southend-on-Sea, United Kingdom

## Abstract

**Aims:**

Older adults experiencing acute mental health crises often face prolonged inpatient stays due to multimorbidity and complex psychosocial needs. The shift from hospital care to supporting people to recover in their own homes, with the right support in place, was the basis for the introduction of the Functional Intensive Response & Response Team (FIRST).

The South-East Essex FIRST (SE FIRST), a virtual ward, which commenced operations in June 2023, was one of three teams set up to provide these services across our trust. It is a multidisciplinary service catering for older adults aged ≥70 years, with flexibility to accommodate 65–69-year-olds with significant frailty. Its objectives were to intervene early, act as a gatekeeper for informal psychiatric hospital admissions, and support timely hospital discharge.

This service evaluation reviewed all referrals from June 2023 to June 2025. Approval was obtained from the Research Team, and data governance principles were adhered to.

**Methods:**

Information was extracted from referral records supplemented by the electronic systems. This included socio-demographic, clinical, and referral variables, which were analysed using descriptive statistics.

**Results:**

In total 309 unique referrals were received, out of which 21 (6.8%) individuals required multiple episodes of care over the period. The majority were female (64.7%), with a mean age of 77.2(±7.0) years. Over three-quarters (76.1%) had a prior psychiatric diagnosis, with mood disorders (41.1%) and organic disorders (19.1%) being the most prevalent. Most referrals originated from Mental Health Liaison Teams (36.2%) and Older Adults CMHT (34.3%). Mental health deterioration was the leading reason for referral (74.1%) followed by post-discharge support (23.3%). Following a two-clinician assessment and multidisciplinary discussion, nearly a third (30.7%) were not considered suitable for FIRST with signposting to appropriate services, 100 (32.4%) were accepted for home treatment, of which 73 were successfully managed with a mean duration of 33.9 (±50.8) days. This translated to an overall admission avoidance rate of approximately 24%. For those whose admission was unavoidable (n=62), they were considered too risky to be managed in the community. Feedback, though limited (16%), was overwhelmingly positive, highlighting professionalism, kindness, and responsiveness which exemplifies our trust motto of ‘We care, we learn, we empower’.

**Conclusion:**

SE FIRST has demonstrated effectiveness in delivering safe, person-centred crisis care for older adults, reducing admissions rates or duration and promoting continuity of care. Future priorities would include strengthening data systems and implementing structured indicators such as tracking admission avoidance rates, quantifying bed-days saved and optimizing service delivery.

